# G Protein-Coupled Receptors in Osteoarthritis

**DOI:** 10.3389/fendo.2021.808835

**Published:** 2022-01-28

**Authors:** Fanhua Wang, Mingyao Liu, Ning Wang, Jian Luo

**Affiliations:** ^1^Yangzhi Rehabilitation Hospital (Shanghai Sunshine Rehabilitation Center), Tongji University School of Medicine, Shanghai, China; ^2^Shanghai Key Laboratory of Regulatory Biology, Institute of Biomedical Sciences and School of Life Sciences, East China Normal University, Shanghai, China; ^3^Department of Oncology and Metabolism, The University of Sheffield, Sheffield, United Kingdom

**Keywords:** osteoarthritis, G protein-coupled receptors, clinical trials, cartilage degradation, OA pain

## Abstract

Osteoarthritis (OA) is the most common chronic joint disease characterized, for which there are no available therapies being able to modify the progression of OA and prevent long-term disability. Critical roles of G-protein coupled receptors (GPCRs) have been established in OA cartilage degeneration, subchondral bone sclerosis and chronic pain. In this review, we describe the pathophysiological processes targeted by GPCRs in OA, along with related preclinical model and/or clinical trial data. We review examples of GPCRs which may offer attractive therapeutic strategies for OA, including receptors for cannabinoids, hormones, prostaglandins, fatty acids, adenosines, chemokines, and discuss the main challenges for developing these therapies.

## Introduction

**Figure 1:**
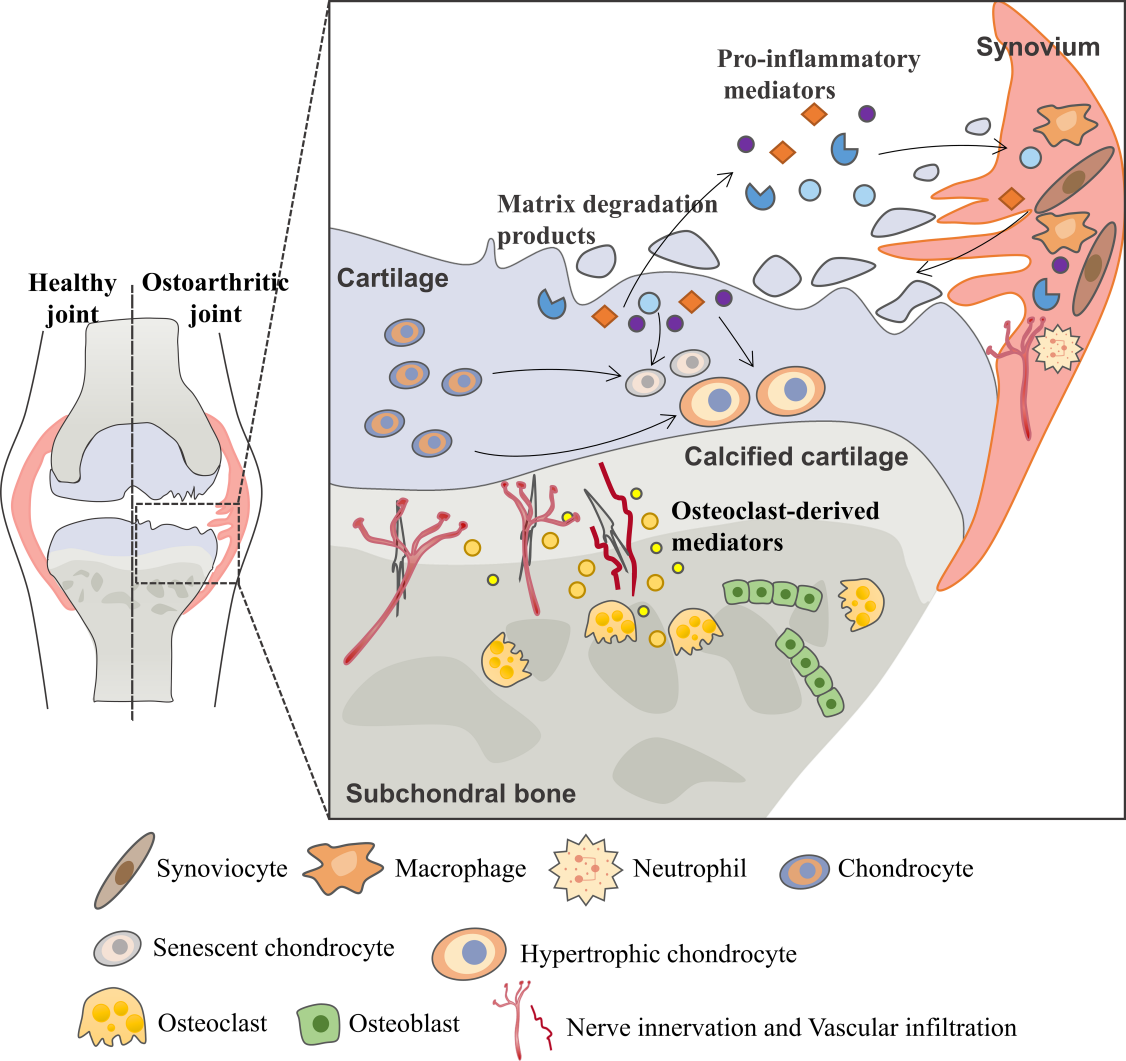
Vicious cycle during OA progression. During the osteoarthritis process, the imbalance between the anabolic activities and catabolic activities of cartilage ECM-degrading enzymes (aggrecanases and matrix metalloproteinases) leads to further extracellular matrix degradation. Products from matrix degradation act on the synovium to induce inflammation and the release of pro-inflammatory mediators (cytokines, chemokines, etc.) that feedback on chondrocyte and cause cartilage breakdown. This process also promotes phenotypic alterations of chondrocytes and leads to chondrocytes hypertrophy and senescence. In the subchondral bone, osteoclasts are activated in response to abnormal mechanical loading and pro-inflammatory mediators, resulting in bone resorption and release of osteoclast-derived mediators which regulate sensory innervation and vascular invasion into the osteochondral junction. This process also correlates with OA pain. Abnormal bone remodeling is then followed by increased bone formation, leading to subchondral bone sclerosis. The homeostatic imbalance of the osteochondral unit increases cartilage susceptibility to disruption and contributes to OA pathological processes.

Osteoarthritis (OA) is the most common degenerative joint disease and one of the leading causes of chronic disability in elderly (1). As a joint degenerative disease, it is characterized by chronic pain, restricted mobility and loss of joint function, increasingly causing a substantial financial burden to society and decreasing quality of life for patients (2). Although OA was primarily thought to be driven by cartilage metabolism disorders, other pathological processes including osteophyte formation, imbalanced subchondral bone remodeling and synovial inflammation are found to form a vicious cycle that contributes to OA progression (**Figure 1**) (3, 4).

Multiple cells, including chondrocytes, osteocytes, osteoclasts, osteoblasts, endothelial cells and sensory neurons, all contribute to this progression (5–7). Early during the cycle, changes first occur in cartilage, including the disruption of chondrocytes pericellular matrix and increased metabolic activity of chondrocytes. As the disease progresses, microscopic cracks are observed in the superficial zone of the articular cartilage, and subchondral bone plate becomes thinner and more porous. With further disease progression, erosion of extracellular matrix (ECM) and increased senescent chondrocytes lead to the development of deep fissures. In the subchondral microenvironment, in response to abnormal mechanical loading and pro-inflammatory mediators, osteocytes upregulate the ratio of RANKL/OPG and osteoclasts are activated resulting in bone resorption and active angiogenesis. In late-stage OA, cartilage chondrocyte death is evident and calcified cartilage expands into the superficial zone of articular cartilage. In addition to the development of subchondral bone cysts, growing sensory innervation and vascular invasion from subchondral bone into cartilage, and osteophyte formation also occur.

Patients with OA experience pain and disability, for which there are predominantly palliative options, such as pain management with analgesics/anti-inflammatory medication and intra-articular injections of corticosteroids (8–10). No current pharmacological therapy is able to exhibit convincing disease-modifying efficacy and prevent long-term disability. Developments in the understanding of OA pathophysiology have enabled the identification of a variety of potential therapeutic targets involved in OA pain, synovial inflammation or structure alteration. Emerging putative disease-modifying OA drugs (DMOADs) hold promise for OA management by regulating cartilage anabolic or catabolic processes, subchondral bone remodeling or synovial inflammation (6, 11). However, the clinical benefit of OA treatments is uncertain as most clinical trials of DMOADs fail to rescue the pathophysiological changes in OA, in addition to the challenges caused by the long follow-up period of clinical trials in developing DMOADs. Therefore, novel OA management strategies are urgently needed.

G protein-coupled receptors (GPCRs), receptors with seven transmembrane domains, comprise the largest and most diverse family of integral membrane proteins that participate in different physiological processes, such as neurotransmission, hormone release, heart contractility and immune responses (12). Based on structural similarities, GPCRs are divided into 6 major families. Only four families are present in humans, including class A (rhodopsinlike) family, class B (secretin) family, class C (metabotropic glutamate-like) and class F (frizzled/smoothened) family. Among them, class A is the largest family with approximately 670 receptors (13, 14). GPCRs couple extracellular stimuli to intracellular responses *via* two main signal transduction mechanisms: heterotrimeric G proteins-dependent and -independent. G proteins are heterotrimeric guanine nucleotide binding proteins that consist of Gα, Gβ and Gγ subunits (15, 16). The coupling specificity and downstream regulation of GPCRs are largely driven by Gα -subunits, which are classified as Gs, Gi/o, Gq and G_12/13_ according to their functions. Gβ and Gγ subunits form a constitutive heterodimer that binds reversibly to the Gα subunit. After activation of GPCRs, Gβγ subunits are released to trigger the activation of downstream effect signaling pathways. These free subunits are competent to interact with the downstream enzymes or channels to drive second messenger generation and modulate cell physiology (17, 18). Once G proteins are released, the protein kinase family of G-protein coupled receptor kinases (GRKs) phosphorylate the intracellular region, after which the phosphorylated GPCRs recruit β-arrestins. This leads to the desensitization and internalization of GPCRs, thereby playing the role of “closing” signal, as a negative feedback of G protein-dependent GPCR signaling. In addition, MAPK and PI3K/Akt signals can be activated by β-arrestins or the Gq pathway, indicating that there is potential crosstalk between heterotrimeric G protein-dependent and independent signaling pathways (19, 20).

**Figure 2:**
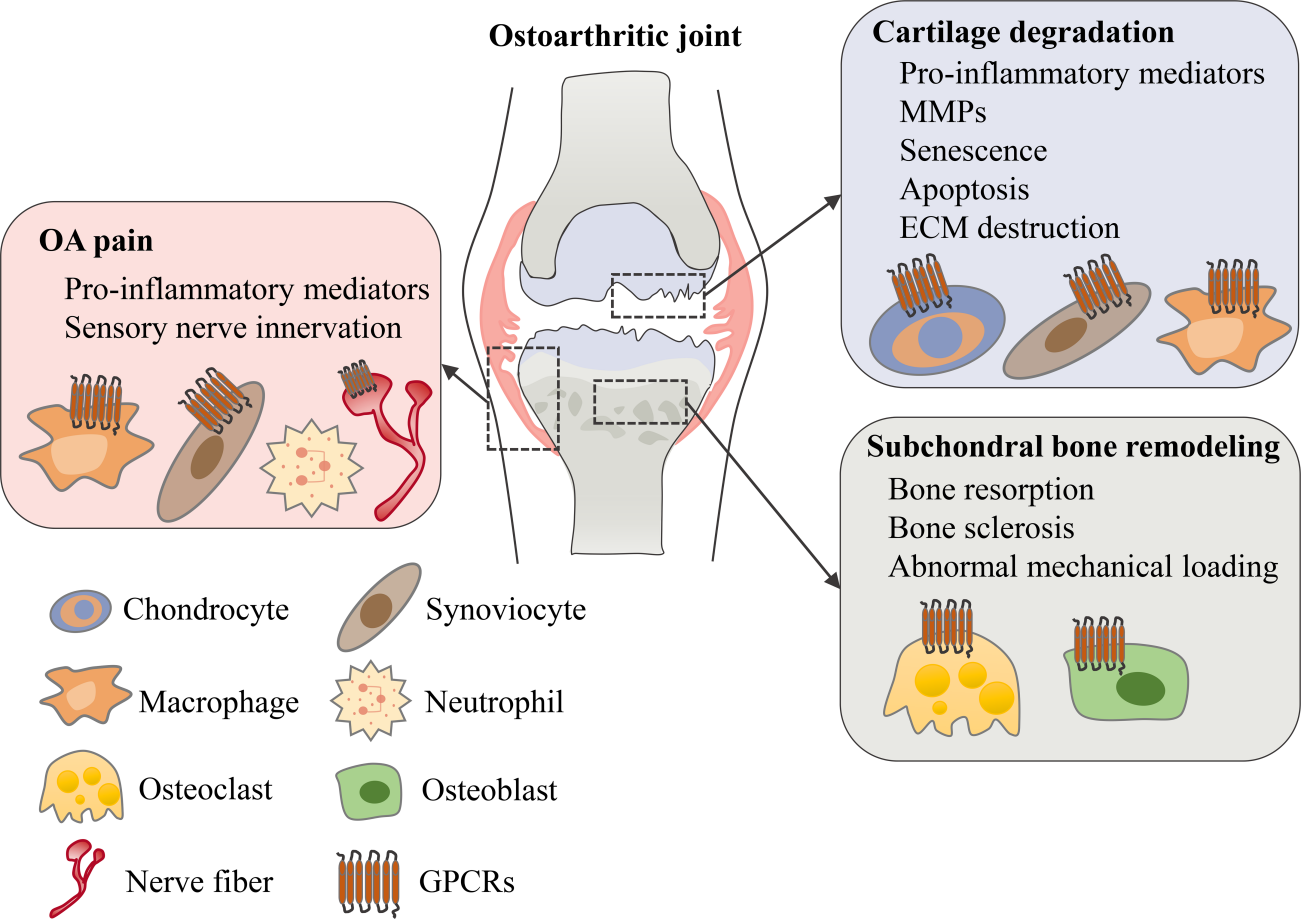
The role of GPCRs in osteoarthritis. In an osteoarthritic joint, GPCRs are expressed in different tissues and cell types. Various types of GPCRs mediate and regulate OA symptoms including cartilage degeneration, subchondral bone remodeling and OA pain.

GPCRs are important targets for drug discovery largely owing to the wide range of physiological and pathophysiological processes in which GPCR targeting can have a major impact. To date, approximately 500 approved drugs target GPCRs, which accounts for almost 30% of all drugs approved by FDA (14, 19). Although most GPCR-targeted drugs are for metabolic diseases, cancers, neurodegenerative diseases and others (21–23), it has been reported that several different types of GPCRs are important for regulating OA symptoms including cartilage degeneration, subchondral bone sclerosis and chronic pain (**Figure 2**). In this review, we’ll review current understanding of these GPCRs’ physiological roles and mechanistic involvements in OA, and discuss emerging therapeutic targets that show promise in preclinical models of OA and/or in clinical trials.

## Cannabinoid Receptors

Over the past decade, the endocannabinoid system has emerged as a potential target for OA therapy with evidence of its involvement in cartilage destruction and OA pain. Cannabinoids target cannabinoid receptors 1 and 2 (CB1 and CB2), two GPCRs originally identified as classical cannabinoid receptors (24, 25). Both cannabinoid receptors have been suggested to be expressed in arthritis tissues including OA cartilage, subchondral bone and synovial tissue (26–28). It has been extensively demonstrated that natural cannabinoids have anti-inflammatory properties and can protect cartilage from degradation during OA (26, 29, 30). ACEA, a CB1 agonist, suppresses interleukin 1 beta (IL-1β)-induced senescence in human primary chondrocytes (31). In a surgical mouse model of OA, the CB2 receptor has been shown to regulate susceptibility to OA. The study revealed that genetic ablation of CB2 aggravated OA pathogenesis compared to wild-type OA mice (32). Additionally, CB2 depleted chondrocytes produced less proteoglycans *in vitro*. Moreover, HU-308 (CB2 agonist) and WIN55212-2 (synthetic cannabinoids) prevented cartilage degradation, while WIN55212-2 was also found to inhibit the activity of an aggrecanase, a disintegrin and metalloproteinase with thrombospondin motifs-4 (ADAMTS-4) (31). These studies suggest that the endocannabinoid system protects against cartilage degradation.

Furthermore, endocannabinoids and their receptors have been reported in osteoblasts, osteoclasts and osteocytes (33–35). CB1 regulates bone growth during skeletal development, while CB2 plays an important role in maintaining the balance between bone resorption and bone formation (36, 37). Knockout of CB2 led to accelerated age-related osteoporosis in mice, while CB1 knockout mice expressed less nuclear factor kappa B ligand (RANKL), suggesting their possible roles in bone remodeling processes during OA (38, 39). CB1 and CB2 receptors are located in synovial tissue where they are expressed on nerve endings that innervate the knee (38, 40). In a monoiodoacetate (MIA) model, an OA model that intra-articular injection of sodium monoiodoacetate induces chondrocyte cell death in the articular cartilage, OA pain and articular structural changes (41, 42), agonists of CB1 (ACEA) and CB2 (JWH133 and A-796260) all decreased pain behavior or subchondral bone degeneration (43–45). However, current clinical trials targeting the endocannabinoid system in OA gave inconclusive results. A randomized clinical trial in OA patients of PF-04457845, a potent FAAH (fatty acid amide hydrolase with a prominent role in the hydrolysis of endocannabinoids) inhibitor, indicated no significant difference in analgesia compared to placebo (NCT00981357) (46). In another on-going phase II study (NCT03098563), patients with knee OA are being treated with combinations of cannabinoids, benzodiazepines, and opioids for evaluating changes in pain ratings and sensitivity but no results have been published yet.

## Chemokines and Receptors

Chemokines and their G protein-coupled receptors control the migratory patterns, positioning and cellular interactions of immune cells, and also induce the recruitment of immune cells into the organs. High levels of chemokines have been observed in rheumatoid arthritis (RA), systemic lupus erythematosus (SLE) and idiopathic inflammatory myopathies (IIM), which are systemic autoimmune disorders (47, 48). Many studies have also found that chemokine system is involved in the process of OA. In this section, we summarize the pathogenic functions of chemokines and their receptors in OA, and discuss their potentials as therapeutic targets.

CXC motif chemokine ligand 12 (CXCL12), also known as SDF-1, is recognized as a homeostatic cytokine. SDF-1 and its receptor C-X-C motif chemokine receptor 4 (CXCR4) and CXCR7 play multiple regulatory roles. SDF-1 is involved in the regulation of cartilage tissue homeostasis and can also regulate chondrocyte proliferation, survival, differentiation (49–51). SDF-1 was shown to negatively regulate mesenchymal stem cell (MSC) chondrogenesis, but the effects of SDF-1 on chondrocyte proliferation and death varied in different studies. CXCR4 and CXCR7, both expressed by chondrocytes, regulate homeostatic and pathological processes during the progression of OA. The upregulated CXCL12/CXCR7 signaling aggravated joint destruction in mice. SDF-1/CXCR4 induced chondrocyte hypertrophy during endochondral bone formation, and the induction of hypertrophic chondrocyte markers, including Runt-related transcription factor 2 (RUNX2), Collagen type X (COLX), and matrix metalloproteinase 13 (MMP13) in chondrocytes, required the presence and interaction of both SDF-1 and CXCR4 (52). During ECM degradation in OA, SDF-1/CXCR4-mediated upregulation of aggrecanase occurred *via* activation of the nuclear factor-κB (NF-κB), mitogen-activated protein kinase (MAPK), and Wnt/β-catenin in chondrocytes (53). Moreover, SDF-1/CXCR4 regulates the crosstalk between subchondral bone and articular cartilage in OA pathogenesis (54). Subchondral bone deterioration and excessive bone resorption were aggravated by increased SDF-1 in anterior cruciate ligament-transection (ACLT) mice. SDF-1 from subchondral bone binds to CXCR4 in chondrocytes and induces articular cartilage degradation by promoting shift of TGF-β receptor 1 (TβRI) signal transduction from activin receptor-like kinase 5 (ALK5) to ALK1. The impact of TGF-β on cartilage is anabolic through ALK5 while catabolic through ALK1 (55, 56). Indeed, AMD3100, a specific inhibitor of SDF-1/CXCR4 axis, attenuated OA by stabilizing subchondral bone microarchitecture and protecting the integrity of cartilage. In addition, studies have demonstrated that TN14003, another antagonist of CXCR4, was more effective in inhibiting release of matrix-degrading enzymes, such as MMP3, MMP9 and MMP13, and in preventing collagen type II (COL2a1) and aggrecan (ACAN) degradation (57, 58). Mechanistically, FGFR3 inhibits CXCR7 expression and CXCL12-dependent macrophage chemotaxis through regulating the NF-κB pathways. FGFR3 deficiency in myeloid cells results in more severe joint destruction and synovitis in the destabilization of the medial meniscus (DMM)-induced mouse OA model and in aging mice, whilst blocking CXCR7 in FGFR3 deficiency mice relieved joint destruction of age-related/DMM-induced arthritis (59). Thus, SDF-1 (as CXCL12) plays an important role in the development of OA and further preclinical and clinical studies are warranted to investigate the feasibility of therapeutically targeting SDF-1/CXCR4/CXCR7 signaling to treat OA.

Other CXCRs may also be involved in the development of OA. For example, even though CXCR2^-/-^ mice do not spontaneously develop arthritis, the blockade of CXCR1/2 signaling led to decreased ECM production and increased chondrocyte apoptosis. These pathological changes result in the loss of phenotypic stability in chondrocytes and promote OA-like phenotypic alternations (60, 61). CXCR3 protein level was also significantly increased in OA patients while knockdown of CXCR3 receptor attenuated chondrocyte apoptosis induced by sodium nitroprusside (62). In the collagenase-induced osteoarthritis (CIOA) model, neutrophils and NK cells were showed to be increased in the synovium as disease-promoting immune cells. The CXCL10/CXCR3 axis promoted the accumulation of NK cells and macrophages in the OA joint, whereas CXCR3^-/-^ mice failed to develop CIOA (63).

C-C chemokine receptor type 5 (CCR5), the receptor for C-C motif chemokine ligand 4 (CCL4) and CCL5, is expressed in normal chondrocytes but at elevated levels in OA chondrocytes. Cartilage degeneration was markedly reduced in CCR5^-/-^ mice affected by post-traumatic OA, while mild changes appeared in osteophyte formation and synovitis compared to wild-types (64). These phenotypes suggest that CCR5 plays a selective role in joint damage.

In the bone microenvironment, CCL2, a key regulator mainly expressed by osteoblasts, promotes subsequent recruitment and migration of mononuclear cells *via* binding to CCR2 (65). Additionally, CCL2 stimulation enhanced the apoptosis of OA chondrocytes while intra-articular injection of CCL2 in mouse knees induced cartilage degradation (66). This result suggests that CCL2/CCR2 axis is involved in cartilage destruction. Further studies showed that CCR2^+^ macrophages were abundant in OA synovium and in association with articular cartilage tissues. Receded OA pathogenesis is accompanied with lessened local macrophage numbers in CCR2- knockout mice. Pharmacological intervention by RS-504393, a CCR2 antagonist, effectively diminished OA disease progression in part by reducing synovial macrophage accumulation (67). In conclusion, disruption of CCL2/CCR2 signaling contributes to reduced macrophage accumulation, synovitis and cartilage breakdown in murine OA models.

Intriguingly, chemokine receptors are critical regulators of neurodegenerative conditions and synapse activity, contributing to pain management. In mice, intra-articular/peripheral tissues injections of CXC chemokines induced pain-like behaviors (68). CCL2/CCR2 signaling was upregulated in the joint innervating dorsal root ganglion. This result was clearly associated with movement-provoked pain behaviors after disease induction. Macrophage infiltration and movement-provoked pain behaviors were not developed in CCR2-null mice. However, CCR2-null mice had similar severe allodynia and structural knee joint damage. These results suggested that targeting the CCL2/CCR2 axis will have clinical benefits for OA pain (69–71). A placebo-controlled, Phase II trial testing PF-04136309 (the specific CCR2 antagonist) for OA pain has been completed but the results are as yet unknown (NCT00689273).

Evidence from pre-clinical studies suggests that the development of more effective inhibitors of chemokine receptors has attractive therapeutic potential in treating OA. It should also be noted that numerous chemokines and their receptors are involved in OA pathogenesis, thus targeting the relevant multiple receptors might be needed for therapeutic benefits.

## Metabolite-Sensing GPCRs

The main metabolite-sensing GPCRs bind metabolites derived from common foodstuffs, including long-chain fatty acids (LCFAs), medium-chain fatty acids (MCFAs), short-chain fatty acids (SCFAs), bile acid, and various others. It has been reported that free fatty acids (FFAs) contribute to skeletal health, as increasing the supplementation of long-chain polyunsaturated fatty acids (LCPUFAs) positively contributes to joint health and prevents osteoporosis (72–74). LCPUFAs are essential factors to support cartilage homeostasis. Studies have revealed that long-chain ω-3 fatty acids reduced secretion or expression of inflammatory cytokines and matrix-degrading enzymes involved in cartilage degradation, such as collagenases or aggrecan-degrading enzymes (aggrecanases). SCFAs augmented systemic bone mass by protecting from bone resorption and suppressing inflammation in chondrocytes (75, 76). In this section, we introduce the metabolite-sensing GPCRs involved, biological relevance between metabolism and osteoarthritis, and highlight the beneficial effects of nutritional protection.

Five GPCRs, including GPR40, GPR41, GPR43, GPR84 and GPR120, have been reported to be activated by FFAs. Among which, GPR40 and GPR120 are receptors for LCFAs, GPR41 and GPR43 for SCFAs, while GPR84 for MCFA. OA progression in the knee joint instability-induced OA model was aggravated in GPR40^-/-^ mice, and GPR40^-/-^ chondrocytes secreted more inflammatory mediators and decreased anabolism upon IL-1β treatment (77). In contrast, GW9508, a GPR40 agonist, blocked degeneration of type II collagen and aggrecan by attenuating the expression of matrix-degrading enzymes and pro-inflammatory cytokines *in vitro* (78). GPR120^-/-^ mice displayed an accelerated progression of ACLT surgery-induced OA (79). GPR120 agonists, GW9508 and TUG891, prevented IL-1β-induced reduction of ECM through SRY (sex-determining region Y)-related HMG (high mobility group) box 9 (SOX9) mediated expression of collagen II and aggrecan in ATDC5 chondrocytes (80). In our previous research, we found that MCFAs receptor GPR84 signaling safeguarded cartilage homeostasis. Activating GPR84 by 6-OAU (agonist) or lauric acid (natural ligand) resulted in increased expression of ECM-related genes in chondrocytes and protected human OA explants against degeneration (81). SCFAs receptors, such as GPR43, were also shown to be involved in chondrocyte homeostasis. Butyrate, a SCFA produced through microbial fermentation in gut, decreased the inflammatory response in IL-1β-stimulated chondrocytes, including reduced expression of pro-inflammatory mediators (cyclooxygenases 2, nitric oxide synthase 2, IL-6), pro-inflammatory adipokines (lipocalin-2 and nesfatin-1), and adhesion molecule (Vascular cell adhesion molecule 1 and Intercellular adhesion molecule 1). Importantly, the anti-inflammatory activities of butyrate were completely dampened by GPR43 silencing (82).

TGR5, a bile acid-sensing GPCR expressed in cultured chondrocytes, showed reduced expression in response to IL-1β/tumor necrosis factor alpha (TNFα)-stimulation in chondrocytes or OA patient chondrocytes. Furthermore, activation of TGR5 using the specific synthetic agonist, INT-777, significantly decreased IL-1β induced senescence and rescued TNFα-induced decreased expression of ECM-related genes in SW1353 cell (83, 84).

These studies of metabolite-sensing GPCRs provide intriguing links between the fields of nutrition, metabolism and OA, which provide insights that nutrient intervention may become new approaches for OA treatment or prevention. The main drawback of research in metabolite-sensing GPCRs and OA is the insufficiency of translational studies using animal models and related clinical trials.

## Adenosine Receptors

Adenosine is a catabolite of ATP and can act as a physiological regulator. Adenosine binds and activates four adenosine receptor subtypes (A1, A2A, A2B, and A3), which are all GPCRs (85, 86). In bone homeostasis, adenosine receptor-mediated mechanisms are involved in bone fracture and repair, and response to loading (87, 88). Articular chondrocytes in humans express primarily A2A receptor (A2AR) and A2B receptor (A2BR) subtypes (89). When cartilage has an aging phenotype or cartilage homeostasis is destroyed, the extracellular ATP will decrease, leading to a decrease in the content of adenosine. Subsequently, the reduction of extracellular adenosine concentration increases the release of chondrocyte-damaging molecules. These molecules include nitric oxide (NO), prostaglandin E2 (PGE2), MMPs, ECM fragments, which further contribute to the cartilage destruction and the pathogenesis of OA (90, 91). Observations in mice deficient of A2AR and ecto-5′ nucleotidase (an enzyme that converts extracellular AMP to adenosine) showed consistent results of developing spontaneous OA. In contrast, intra-articular injection of adenosine prevented development of OA and restored the cartilage homeostasis by engaging A2AR in rats (92). This could be due to the fact that the exogenous adenosine activates A2AR and regulates the pathogenesis of OA *via* suppressing the expression of a variety of pro-inflammatory cytokines, such as NO, PGE2, IL-1 and TNF. The anti-inflammatory role of A2AR has indeed been proposed in mouse articular chondrocytes treated with hyaluronan oligosaccharides or collagen-induced arthritis (CIA) (93, 94). In addition, studies have shown that A2AR stimulation enhances mitochondrial metabolism and prevents mitochondrial injury. Intra-articular injections of a liposomal A2AR agonist improved the reactive oxygen species (ROS) burden, proteoglycan catabolism and chondrocyte viability in knee cartilage of obesity-induced OA mice (95). Moreover, polydeoxyribonucleotides (PDRNs), deoxyribonucleotide polymer chains with 50-2000 base pairs in length, can counter proteoglycan degradation in cartilage explants by decelerating the activity of MMPs (96) and can also activate A2AR to decrease cytokine production and reduce cartilage erosion of collagen-induced arthritis (97). There have been multiple randomized, double-blind clinical trials comparing the efficacy of intra-articular polynucleotides and hyaluronic acid injections in treating knee osteoarthritis. Results suggested that Knee Society Score total score (KOOS) and pain items were statistically significantly ameliorated in both polynucleotides- and hyaluronic acid-supplemented groups, with higher efficacy in the polynucleotides group. Additionally, polynucleotides led to significant symptomatic relief as measured by the KOOS after only 2 weeks of treatment, while similar improvements with administration of hyaluronic acid were seen after 18 weeks (98–101).

Other adenosine receptor subtypes have also been suggested to have potential roles in OA. The A2BR has been associated with chondrogenic differentiation. A2BR agonists suppressed hMSC chondrogenic differentiation through downregulating the expressions of ECM-related genes and cartilaginous transcription factors, while antagonists of A2BR induced chondrogenic differentiation of hMSC (102). Ablation of A3R led to development of OA in aged mice. A3R selective agonists protected cartilage by downregulating intracellular CaMKII kinase and RUNX2 transcription factor (103). CF101, a highly selective, synthetic agonist to the A3R, can induce apoptosis of inflammatory cells, and prevent cartilage damage and bone destruction in rat knee osteoarthritis (104). It is worth noting that excessive adenosine supplement to body is not advisable, as children lacking adenosine deaminase develop chondrodysplasia, with plasma adenosine levels increasing to the micromolar level (105, 106). In summary, the adenosine receptor is an important homeostatic regulator of cartilage homeostasis, cartilaginous inflammation and OA progression. Therefore, adenosine supplement may represent a novel approach for OA treatment.

## Protease-Activated Receptor

Proteinase-activated receptors (PARs) constitute a unique family of GPCRs that are widely expressed by fibroblast-like cells, chondrocytes and osteoblasts, immune cells in joints as well as in sensory neurons. Proteolytic enzymes signaling *via* PARs have been implicated in inflammation and pain in RA. For a comprehensive review, please refer to Oikonomopoulou et al., 2018 (107). PAR2 was detected in chondrocytes and synovial tissues from OA patients, while expression of PAR2 in OA chondrocytes was upregulated by IL-1β/TNFα (108, 109). Activation of PAR2 in human OA cartilage upregulated catabolic and pro-inflammatory pathways, resulting in cartilage degradation (110). PAR2 expression was significantly upregulated in articular cartilage in OA mice. Several studies suggested that deletion of PAR2 retarded the OA progression, cartilage damage, and subchondral bone remodeling disequilibrium in OA mouse models (111–113). Additionally, PAR2 has been shown to be expressed in the proliferative/hypertrophic chondrocytes within osteophytes. PAR2^-/-^ mice presented less osteophyte formation, no osteosclerosis, and reduced pain perception in a DMM model. Intra-articular injection of hPAR2 in PAR2^-/-^ mice restored osteophyte formation and cartilage damage to the similar level as in wild-type mice, confirming the pathogenic role of PAR2 in the DMM model (114). Further studies showed that AZ3451, an antagonist of PAR2, prevented the IL-1β-induced inflammatory cytokines release, catabolic gene expression, senescence, and apoptosis in chondrocytes. Intra-articular injection of AZ3451 ameliorated cartilage destruction in a rat OA model (115). Therefore, PAR2 has the potential to orchestrate OA-related pain, cartilage and bone pathology. It is plausible that, through further preclinical and/or clinical verification, targeting proteolytic pathways can bring in benefits to RA or OA patients and reduce joint damage and inflammation.

## Prostaglandin Receptors

PGE2, the most abundant prostaglandin in most tissues, is generated by the initial actions of the cyclooxygenases on arachidonic acid (116). COX-2-selective non-steroidal anti-inflammatory drugs (NSAIDs) reduce pain and inflammation, and are thought to act *via* inhibiting PGE2 in humans (117, 118). The cartilage releases a high level of PGE2, a key pro-inflammatory and joint pain molecule in OA patients. PGE2 binds to four specific G protein-coupled receptors, prostaglandin E receptor 1-4 (EP1-4). Among them, EP2 and EP4 have been found to be associated with cartilage repair and OA development. Early research showed that simultaneous stimulation of EP2 and EP4 enhanced proteoglycan accumulation and intracellular cyclic adenosine 3’,5’-monophosphate (cAMP) production during the differentiation of rat primary chondrocytes (119). The expression patterns of EP2 and EP4 are different during the commitment of MSC to chondrogenesis. EP4 expression continuously increases in this process, while the expression of EP2 increases at the earlier stage and then decreases (120). Other studies showed that growth-promoting and apoptosis-protecting genes were upregulated in human articular chondrocytes treated with EP2 agonists. The culture of rat femurs showed an increase of proliferating cell nuclear antigen (PCNA) staining in chondrocytes, suggesting EP2 enhanced the growth in articular cartilage (121). Gelatin hydrogel containing PLGA microspheres conjugated with ONO-8815Ly, a selective EP2 agonist, promoted tissue regeneration in a rabbit chondral and osteochondral defect model (122), whilst intra-articular injections of EP2 agonist lessened joint pain and promoted tissue repair of osteochondral defect in rabbits (123). Furthermore, an EP2 agonist enhanced reconstruction of the boundary between articular cartilage and subchondral bone, which is imperative to maintain the articular structure. It is interesting to note that the regenerated tissue contained both EP2- and PCNA-positive chondrocytes, indicating that the cartilage regeneration was executed mainly by EP2-positive cells (122). The same research team also found that ONO-8815Ly prevented cartilage degeneration in ACLT and DMM-induced cartilage traumatic models, which was associated with restraining the expression of MMP13, a catabolic factor to matrix network (124). Similarly, another study demonstrated EP2 agonist downregulated MMP13 mRNA expression *via* the cAMP- protein kinase A pathway in osteoarthritis chondrocytes (125).

A previous report suggested that EP4 was upregulated in OA cartilage. However, effects of EP4 on the cartilage catabolism during OA progression still remain controversial. EP4 antagonist (AH23848) prevented PGE2 induced matrix degradation and MMP13 expression in OA cartilage explants, implicating EP4’s pivotal role in mediating the PGE2 catabolic effects during OA progression (126). To the contrary, another study showed that PGE2 inhibited IL-1β-induced expression of MMP1 and MMP13 *via* EP4 by suppressing MKK4-JNK MAPK-c-JUN pathway in human chondrocytes (127). Furthermore, the EP4 receptor mediates the PGE2-elicited inflammation and sensitization of sensory neurons, while EP4 inhibition contributes to the development of targeted therapies for anti-inflammatory and analgesic effect in OA (128–131). Grapiprant, an EP4 antagonist, has been approved for by the FDA treating OA pain in dogs (132, 133). A multicenter, randomized study demonstrated that the inhibitor of microsomal prostaglandin E synthase-1 (LYA) but not the EP4 antagonist (LYB) improved clinical signs of OA pain in dogs (134). Although there are animal model studies and clinical applications in effects of EP receptors in OA, the *in vivo* functions and molecular mechanisms of EP receptors in cartilage homeostasis and OA need further investigation. In particular, there is no relevant research using gene-level ablation of EPs to verify their functions in cartilage, while conditional knockout mice should be considered in order to avoid the lethal consequence of genome-wide knockout.

## Hormone Receptors

Hormone receptor signal transduction, such as for norepinephrine (NE) and epinephrine, plays important roles in articular cartilage homeostasis and OA. In this section, we summarize the relevant research on hormone receptors involved in the cartilage system. α2A- and β2-adrenoreceptor positive chondrocytes were observed in cartilage, with more evidence in the pre-hypertrophic and hypertrophic cartilage. Intercepting α2A-adrenoreceptor increased aggrecan production and decreased MMPs expression in the degraded temporomandibular joint cartilage of rats (135, 136). NE reversed IL-1β induced production of IL-8, MMP13, COL2, and glycosaminoglycans, and decreased proliferation in chondrocytes. This was achieved *via* β-adrenoreceptor signaling. However, NE was also shown to increase proliferating cells and induce apoptosis *via* α1- adrenoreceptor in chondrocytes (137).

The calcitonin receptor was identified in bovine articular cartilage (138). KBP, an agonist of amylin and calcitonin receptors, counteracted DMM induced cartilage erosion, degradation biomarkers and pain behavior in rats (139). Nerves containing the calcitonin gene-related peptide (CGRP) have been implicated in a number of pain scenarios. The CGRP release has been observed in the joints of OA rodents, as perivascular sensory and sympathetic nerve fibers innervate the osteochondral junction in osteoarthritic knees (140–142). Innervation of CGRP^+^ neurons in subchondral bone was significantly augmented after OA induction, whilst blockade of CGRP^+^ sensory fibers innervating in the subchondral bone reduced OA pain (143, 144). In addition, antagonizing the CGRP receptor ablated mechanosensitivity of joint nociceptors in MIA and DMM OA rats (145).

The parathyroid hormone (PTH)/parathyroid hormone-related protein (PTH/PTHrP) receptors are well known for their biologic actions in controlling mineral homeostasis, bone development, and bone remodeling (146–148). Additionally, activation of the PTH/PTHrP receptor slowed the chondrocyte proliferation and delayed chondrocyte hypertrophy (149, 150), although other studies showed that PTHR1 is upregulated in OA cartilage (149, 151). Importantly, teriparatide (recombinant human PTH), an FDA-approved treatment for osteoporosis, has been shown to decelerate cartilage degeneration and induce matrix regeneration in post-traumatic osteoarthritis mice (152). Currently, a randomized clinical trial attempting to evaluate teriparatide as a chondroregenerative therapy for OA is ongoing (NCT03072147). This could present a new promising clinical application for the drug by re-purposing it for OA treatment.

Several other hormone receptors were also detected to be expressed in the cartilage tissue or chondrocytes, which may indicate novel targets. For instance, follicle stimulating hormone receptor (FSHR) was detected in mouse chondrocytes and human cartilage tissue (153). Oxytocin receptor (OTR) was expressed in human primary chondrocytes, and significantly reduced in OA chondrocytes (154). Angiotensin II receptor (ATIIR) affected the proliferation and apoptosis of chondrocytes under oxygen-glucose deprivation (155). Activation of melanocortin receptor MCR1 and MCR3 downregulated IL-1β, IL-6 and IL-8 release, MMPs expression and inhibited cell death in chondrocytes (156). MCR1-deficient mice developed a more severe OA pathology of cartilage degradation (157). Glucagon-like peptide-1 (GLP-1) is an incretin hormone that activates GLP-1R to regulate glucose and energy homeostasis. Exendin-4, a GLP-1R agonist, alleviated chondrocyte apoptosis and ECM degradation in ACL rats (158). Endothelin receptors ETA and ETB were also expressed in rat chondrocytes (159).

## Other GPCRs in OA

There are also some other GPCRs involved in OA which may represent potential targets and will be briefly summarized in this paragraph. The calcium-sensing receptor (CaSR), senses changes in serum Ca^2+^ in parathyroid glands to regulate PTH. It has been established that knocking out CaSR in chondrocyte prevented matrix degradation in the cartilage of OA mice (160). Frizzled class receptor 4 (FZD4) was shown to be involved in the pathogenesis of temporomandibular joint osteoarthritis, when mediated by miR-101a-3p (161). It has been shown that activation of Kappa opioid receptor (KOR) by chemical agonist U-50,488H inhibited inflammation in arthritic conditions, and KOR^-/-^ mice exhibited accelerated cartilage degeneration in cartilage and subchondral bone defects compared with WT mice (162, 163). Extensive studies have indicated that inflammatory diseases decreased the pH of the cartilage environment (164–166). Acid sensing plays an essential role for maintaining cell function through acid sensing ion channels or proton-activated GPCR (167, 168). The proton-activated GPR4 regulates OA pathogenesis *via* modulating CXCL12/CXCR7 signaling, and inhibition of GPR4 with NE52-QQ57 ameliorates OA development in both mouse models and human articular cartilage explants (169).

## G Protein-Coupled Receptor Kinase in OA

There are seven G protein-coupled receptor kinase (GRKs) subtypes, relevant to the role in GPCR phosphorylation and desensitization, and also phosphorylation of a number of intracellular signaling proteins. Studies demonstrated that GRK5 regulated cartilage degradation in OA progression *via* NF-κB signaling. Intra-articular injection of amlexanox (a selective GRK5 inhibitor and a candidate for OA treatment) protected mouse cartilage against cartilage degradation and reduced the expression of catabolic factors in DMM-induced OA mice (170). Cartilage-specific GRK2 deletion promoted matrix regeneration and prevented OA progression. Furthermore, the GRK2-inhibiting antidepressant paroxetine decelerated OA progression in DMM mice (171). As a clinically used antidepressant with known pharmacological profiles and safety record, paroxetine offers a promising therapeutic strategy for OA that can be easily translated from bench side to clinics.

## Perspectives and Conclusions

Evidence from preclinical models of OA and/or clinical trials have highlighted multiple GPCRs as novel therapeutic targets in OA treatment, and showed promising efficacy in managing OA pain and structural progression (**Table 1**). For instance, the prominent role in multiple arthritis has rendered the adenosine receptor as a promising target for therapeutic intervention. Particularly, results of clinical trials with polynucleotides in OA patients have been encouraging. Interestingly, the fate of MSCs towards chondrogenesis and osteogenesis can be significantly mediated by adenosines *via* ecto-5′-nucleotidase/CD73 through activation of A2AR and A2BR receptors, differentially and respectively (172, 173). With this strategy, MSCs for cartilage and bone repair in damaged parts can be adjusted by regulating the activity of A2AR/A2BR at different stages of joint repair. Metabolite-sensing GPCRs could be an interesting target for OA prevention and treatment, but preclinical animal studies and clinical trials are lacking at this time.

**Table 1:** GPCRs relevant to OA.

GPCR	Cellular function	Pathogenic function in OA	Clinical trials	Agonists/antagonist	Refs
**Cannabinoid receptors**					
CB1	Suppress chondrocyte senescence	Inhibit OA pain	NCT00981357	ACEA	(26–46)
		Decrease subchondral bone degeneration	NCT03098563	HU-308	
				WIN55212-2	
CB2	Promote chondrocyte proteoglycans	Prevent cartilage degradation		A-796260	
				JWH133	
				PF-04457845	
**Chemokines and receptors**					
CXCR4	Induce chondrocyte hypertrophy	Induce cartilage degradation		AMD3100	(49–59)
				TN14003	
CXCR7	Enhance macrophage chemotaxis	Aggravate joint destruction			
CXCR1/2	Increase ECM production				(60, 61)
	Decrease chondrocyte apoptosis				
CXCR3	Increase chondrocyte apoptosis	Aggravate cartilage damage			(62, 63)
	Promote immune cells inflammatory response	Increase synovitis			
		Increase osteophyte formation			
CCR5	Maintain the inflammatory process	Induce cartilage degeneration			(65)
CCR2	Enhance chondrocyte apoptosis	Aggravate cartilage degradation	NCT00689273	RS-504393	(66–71)
	Macrophage infiltration	Increase synovitis		PF-04136309	
		Increase OA pain			
Metabolite-Sensing GPCRs*					
GPR40	Reduce chondrocyte inflammatory	Reduce chondral calcification		GW9508	(77, 78)
	Inhibit chondrocyte catabolism	Reduce osteophyte formation			
		Reduce subchondral bone sclerosis			
GPR120	Protect ECM production	Prevent cartilage degradation		TUG891	(79, 80)
		Reduce synovitis		GW9508	
		Reduce subchondral bone structural change			
GPR84	Increase ECM production	Prevent cartilage degradation		6-OAU	(81)
	Inhibit chondrocyte catabolism	Reduce osteophyte formation			
		Reduce subchondral bone sclerosis			
GPR43	Decrease chondrocyte inflammatory				(82)
TGR5	Decrease chondrocyte senescence			INT-777	(83, 84)
	Protect ECM production				
Adenosine receptors*					
A2AR	Suppress chondrocyte inflammatory	Prevent cartilage degradation		PDRNs	(89–101)
	Enhance mitochondrial metabolism	Reduce synovitis			
	Suppress chondrocyte catabolism	Reduce subchondral bone structural change			
A2BR	Suppress chondrogenic differentiation				(102)
A3R	Induce inflammatory cells apoptosis	Prevent cartilage degeneration		CF101	(103, 104)
		Prevent bone destruction			
**Protease-activated receptor**					
PAR2	Promote chondrocyte apoptosis	Aggravate cartilage damage		AZ3451	(108–115)
	Promote chondrocyte senescence	Increase subchondral bone remodeling			
	Promote chondrocyte inflammatory	Increase osteophytes formation			
	Promote chondrocyte catabolism	Promote OA pain			
**Prostaglandin receptors**					
EP2	Enhance chondrocyte differentiation	Increase cartilage regeneration		ONO-8815Ly	(119–125)
	Protect chondrocyte apoptosis	Lessen Joint pain			
		Prevent cartilage degeneration			
EP4	Chondrocyte catabolism	Matrix degradation		AH23848	(126–133)
	Inflammation	Synovitis		Grapiprant	
		OA pain			
**Hormone receptors**					
α2A-adreno-receptor	Decrease chondrocyte metabolism	Prevent cartilage degeneration			(135, 136)
	Inhibit chondrocyte inflammatory	Prevent subchondral bone loss			
β-adreno-receptor	Protect chondrocyte proliferation				(137)
	Inhibit chondrocyte catabolism				
	Protect ECM production				
PTH/PTHrP receptor	Slow chondrocyte proliferation	Decelerate cartilage degeneration	NCT03072147	teriparatide	(149–152)
	Delay chondrocyte hypertrophy	Induce matrix regeneration			
ATIIR	Affect chondrocyte proliferation and apoptosis				(155)
MCR1	Inhibit chondrocyte catabolism	Prevent cartilage degradation			(156, 157)
		Prevent bone remodeling			
GLP-1R	Alleviate chondrocyte apoptosis	Decelerate ECM degradation		Exendin-4	(158)
**Other GPCRs in OA**					
CaSR	Inhibit chondrocyte differentiation	Aggravate cartilage degradation			(160)
	Inhibit ECM production				
KOR	Increase lubricin production	Prevent cartilage degeneration		U-50,488H	(162, 163)
	Increase GAG synthesis	Prevent subchondral bone defect			
		Prevent joint inflammation			
GPR4	Inhibit ECM production	Aggravate cartilage damage		NE52-QQ57	(169)
	Upregulate Chondrocyte inflammatory and catabolism	Increase synovitis			
		Increase osteophytes formation			

*Promising target for OA treatment and prevention.

In addition to identifying promising drugs for OA management, a well-integrated drug platform incorporating nanocarriers and tissue engineering could provide additional benefits in the treatment of OA. Nanocarriers with a chondrocyte-specific aptamer have been widely used for sustained delivery in cartilage tissue, providing improved targeting specificity and pharmacokinetic profile (174–176). Tissue engineering can lead to the construction of a ‘native’ microenvironment to deliver drug/growth factors, maintain ECM deposition and support mechanical properties as naïve cartilage (177, 178). This integration may form new approaches to the prevention and treatment of OA.

On paper, many of the pathways can be selectively and potently targeted, offering exciting opportunities for OA management. However, it should be noted that complex pathogenic mechanisms of OA limit clinical applications for OA patients. Thus, future research should be directed towards elucidating how these different pathways interact to that drive structural progression or OA pain. Moreover, heterogeneity in clinical presentation and histopathology can make it difficult to elucidate OA pathophysiological changes. In a study published last year, OA patients were classified into four distinct osteoarthritis subtypes with a knee joint tissue transcriptome atlas: a glycosaminoglycan metabolic disorder subtype (C1), a collagen metabolic disorder subtype (C2), an activated sensory neuron subtype (C3), and an inflammation subtype (C4) (179). This provides a new paradigm for precision medicine in the diagnosis and treatment of OA, although they may contradict traditional OA diagnosis by clinical and radiographic presentation. Hence, research that defines meaningful OA phenotypes will be critical in determining optimal treatment strategy, and should be prioritized.

In this review, we have described the pathophysiological processes targeted by GPCRs in OA, along with related preclinical models and/or clinical trials data, and discussed the main challenges and developments for these potential therapies. Further studies are warranted to confirm the translatable symptomatic and long-term benefits of candidate drugs. Meanwhile, expanding the knowledge of the pathophysiological roles of agonists, antagonists or autoantibodies for GPCRs will shed light on the biology of these receptors and provide new insights for potential therapeutic approaches.

## Author Contributions

FW wrote the article. All authors made a substantial contribution to discussion of content and reviewed or edited the manuscript before submission.

## Funding

This work was supported by grants from the Fundamental Research Funds for the Central Universities (22120210586 to JL), Ministry of Science and Technology of China (2020YFC2002800 to JL), the National Natural Science Foundation of China (91949127 to JL).

## Conflict of Interest

The authors declare that the research was conducted in the absence of any commercial or financial relationships that could be construed as a potential conflict of interest.

## Publisher’s Note

All claims expressed in this article are solely those of the authors and do not necessarily represent those of their affiliated organizations, or those of the publisher, the editors and the reviewers. Any product that may be evaluated in this article, or claim that may be made by its manufacturer, is not guaranteed or endorsed by the publisher.
